# Lessons learned: enhancing rural risk communication for future health crises through the PHERCC framework

**DOI:** 10.3389/fpubh.2025.1594833

**Published:** 2025-06-03

**Authors:** Abimbola Leslie, Elizabeth K. Woods, Eline M. van den Broek-Altenburg, Gary S. Stein, Jan K. Carney

**Affiliations:** Larner College of Medicine University of Vermont, Burlington, VT, United States

**Keywords:** stakeholder engagement, health equity, PHERCC framework, public health emergency, risk communication, public health messaging, health communication

## Abstract

**Introduction:**

Public health emergencies, such as the COVID-19 pandemic, highlight the critical role of effective risk communication in managing crises. The Public Health Emergency Risk and Crisis Communication framework (PHERCC) provides a structured approach to crafting, delivering, and refining public health messages to build trust, promote compliance, and enhance societal resilience.

**Methods:**

This qualitative study examined COVID-19 risk communication strategies in rural Northern New England using the PHERCC framework. Data were collected through seventeen stakeholder interviews, seven focus groups, and a pilot study conducted between November 2022 and March 2023. Stakeholders represented state and local organizations, while focus group participants included rural residents. A thematic analysis using NVivo software aligned findings with the six PHERCC domains: Evidence, Initiator, Channel, Publics, Message, and Feedback.

**Results:**

Stakeholders emphasized transparency in public health messaging, adapting to evolving evidence while maintaining consistency. Trusted local sources and traditional media were essential for reaching vulnerable populations, particularly older adults in rural areas. Public feedback highlighted barriers such as misinformation, translation challenges, and limited internet access. The pilot study confirmed that community collaboration and tailored messaging increased understanding and trust among rural residents. Simplified accessible core messages and consistent updates further enhanced public engagement.

**Conclusion:**

This study shows the importance of evidence-based, adaptive, and population subgroup sensitive communication during public health emergencies. The PHERCC framework proved instrumental in addressing challenges, promoting trust, and refining strategies. Investing in inclusive communication systems and leveraging community partnerships are important for effective responses to future health crises.

## 1 Introduction

Communicating risk is an essential tool in responding to public health emergencies, including facilitating and supporting behavior change. Clear and effective risk communication is necessary for improving the public’s understanding of health threats and helping them make informed decisions to reduce potential risks (1–5). The COVID-19 pandemic caused approximately 14.83 million deaths globally to date (6). Despite emergency preparation plans, public health officials and healthcare providers faced significant challenges in addressing the pandemic. During this time, trusted public health leaders faced numerous challenges, especially the task of risk communication efforts to provide guidance on public health measures accessible to population specific groups often under tight timeframes, with uncertain outcomes, and in formats that were difficult to convey (1).

Risk communication is the real-time exchange of information, guidance, and perspectives between decision-makers, experts, and populations exposed to a hazard or imminent threat to their survival, health, or economic and social well-being (7, 8). Its goal is to help those at risk make informed decisions to reduce the impact of hazards and take appropriate protective actions (7). Effective and accessible communication strategies during public health crises, such as the COVID-19 outbreak, are essential for successful preparedness, response, and recovery efforts (9, 10).

It was important during the COVID-19 pandemic for risk communication to inform and persuade individuals and community members with guidance and confidence to adopt protective behaviors. Sharing accurate information promptly, in languages and through understandable, trusted, and accessible channels, enables individuals to make informed evidence-based decisions to protect themselves, their families, and their communities (7, 11–13). Effective communication must instruct, inform, and motivate self-protective behavior; provide timely updates on risks; build trust in officials; and dispel theatrical rhetoric and rumors (7).

Public health outbreaks are characterized by unpredictability and unclear boundaries, emphasizing the role of effective risk communication in shaping robust public health response strategies (9). In public health emergencies, such as pandemics, risk communication aims to improve health outcomes by engaging effectively and delivering essential health information to community specific and vulnerable groups (14–16).

Lessons from past public health crises reveal several critical insights about risk communication. Failures in communication expose systemic limitations in public health response. For example, China’s delayed and inefficient handling of the 2003 SARS outbreak and similar shortcomings in Indonesia’s COVID-19 communication strategy show how gaps in surveillance and risk messaging can erode trust and slow containment efforts (17, 18). These cases emphasize that effective communication must be supported by timely data sharing and strong public health infrastructure. Another key takeaway is the importance of providing high-quality, contextualized information. A study of risk communication during the Ebola outbreak found that while messages were generally factual, they often lacked the context needed to help people accurately assess their risk (19). Messages that included actionable steps were more effective, reinforcing the idea that clear, specific recommendations help reduce fear and increase self-efficacy (19). This pattern has been observed in other crises as well, where ambiguous or incomplete information contributed to public anxiety (20, 21).

A consistent issue across outbreaks is the challenge of communicating under uncertainty. During the Zika outbreak, limited knowledge about transmission and risks to pregnant women created confusion and heightened public concern (20, 21). Officials had to encourage protective actions even as guidance changed, using a variety of approaches, including social media campaigns, to manage public understanding (22, 23). This reflects a broader lesson about the need to acknowledge uncertainty while still offering clear guidance. Together, these examples highlight overlapping lessons: the need to communicate clearly even in the face of uncertainty, the value of actionable and contextual information, and the risks posed by inadequate coordination and transparency.

These lessons underscore the need for structured frameworks to guide communication efforts during public health emergencies. Numerous studies have highlighted the challenges of risk communication in such contexts, including scientific uncertainty, misinformation, distrust in authorities, and ethical concerns like inequality and stigma (24–27). Communication specialists use evidence-based research to assess risk perception, support policymaking, and develop practical strategies for effective risk and crisis management (28).

To support a more systematic communication response, public health disasters often follow a “drop-loop model,” progressing from a baseline to recovery and development, interrupted by a decline triggered by critical events, followed by an acute crisis and stabilization (29, 30). The drop-loop model assists stakeholders and key actors in assessing the situation during an acute public health emergency, helping them identify the challenges faced by affected populations while also anticipating the next phase of the crisis. Because communication needs change at each stage, from the urgency of acute response to the reassurance and trust-building required during recovery, risk communication strategies must evolve accordingly (28).

**Figure 1:**
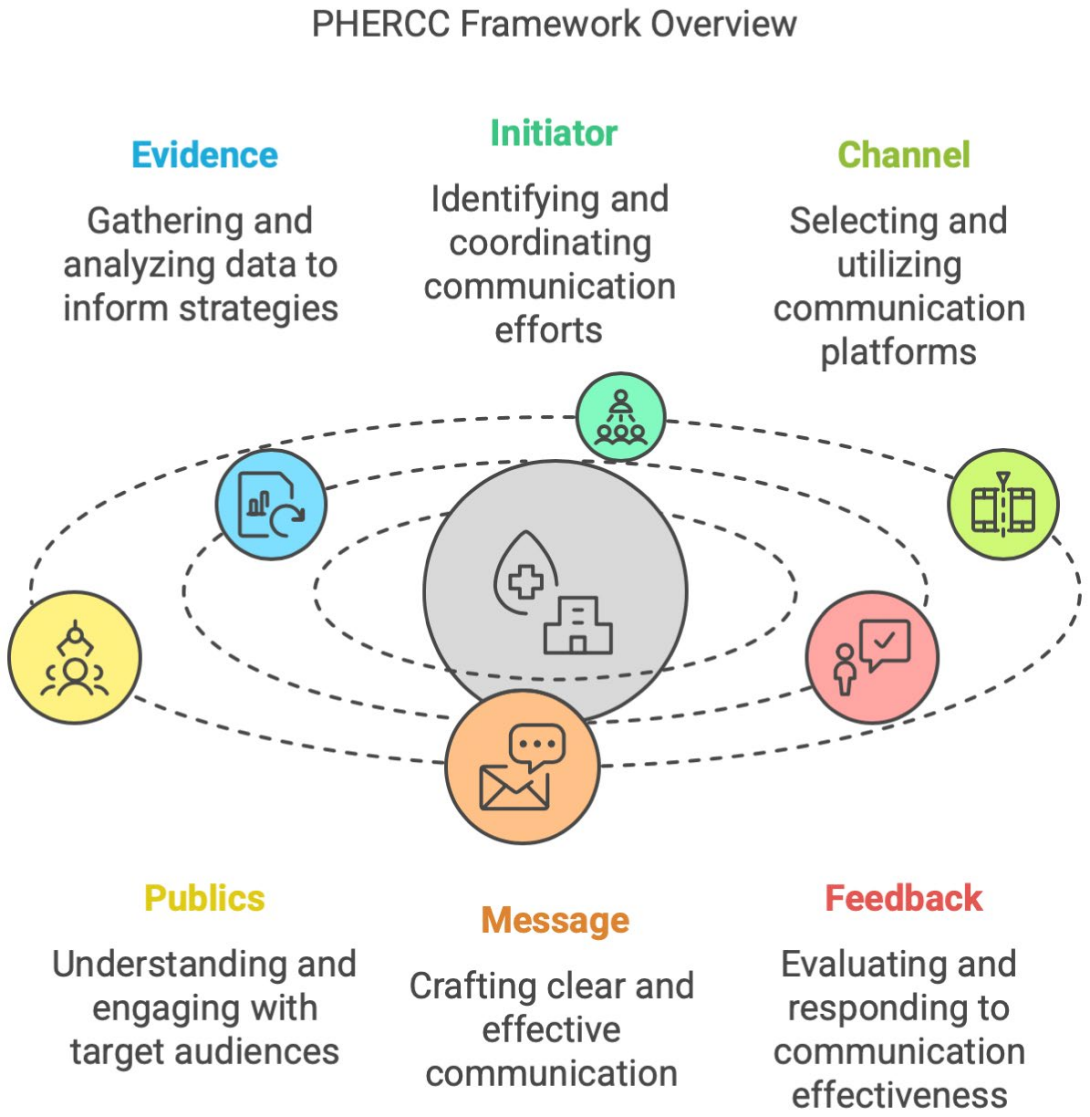
PHERCC framework.

Guiding communication efforts through the various phases of the drop-loop model can be applied in the context of the Public Health Emergency Risk and Crisis Communication (PHERCC) framework (31). The PHERCC framework is an ethical model designed to guide the development, governance, and evaluation of risk and crisis communication strategies in public health emergencies (31, 32). While models such as the CDC’s Crisis and Emergency Risk Communication (CERC) framework also emphasize timeliness, credibility, and empathy, PHERCC expands upon these principles by embedding ethical considerations and equity as central organizing values. It places a stronger emphasis on inclusive governance, bidirectional feedback, and tailoring messages to structurally marginalized communities, which is especially relevant in rural or underserved settings (31). PHERCC plays a crucial role in preparedness and public health response efforts, being deeply integrated into these activities. It supports the development and planning of future response strategies for public health crises, as recent pandemic evidence highlights the moral and practical necessity of being ready for the next emergency. The PHERCC framework is organized into six domains: Evidence, Initiator, Channel, Publics, Message, and Feedback (Figure 1) (33).

This framework posits that the initiator must understand the identity and traits of different audiences, while these audiences, or the public, must also understand and become a basis for confidence in the initiator’s identity and characteristics. The initiator collects all relevant evidence to craft a clear and effective message, tailored to each audience based on its specific needs. The initiator establishes and maintains a strong communication infrastructure or channels, for each audience, develops a series of messages, and, finally, listens to and integrates feedback from each audience as vital input for future communication efforts (34, 35).

The PHERCC framework provides a useful lens for understanding how public health messages were crafted and received during COVID-19, particularly in rural regions where infrastructure and trust vary widely. This paper aims to address the gap in understanding public health emergency risk communication during the COVID-19 pandemic in rural Northern New England. Although prior research has highlighted the disproportionate health and social impacts of COVID-19 on rural communities, including challenges related to limited healthcare infrastructure, digital divides, and socioeconomic vulnerability (36, 37), there has been comparatively less focus on how public health risk communication functioned in these contexts. Rural communities often face distinct barriers to receiving timely, trusted, and culturally appropriate health information (38). This study contributes to the growing literature by exploring how risk communication was experienced and adapted in rural Northern New England. Using the PHERCC framework, we examine both systemic challenges and community-informed solutions. By centering rural perspectives, this research adds nuance to national narratives and helps inform more equitable communication strategies in future public health emergencies.

## 2 Methods

This study employed qualitative methods, beginning with semi-structured interviews with state and local organizational leaders to examine the communication strategies, policies, and programmatic context surrounding COVID-19 testing in a rural Northern New England setting. Findings from these interviews informed the development of focus groups with rural residents. A pilot study was conducted among older adults to collect feedback on health communication materials and identify their information needs and preferences. The findings from this pilot study directly informed our analysis within the PHERCC framework feedback domain.

All stakeholder and focus group procedures and materials were reviewed by the University of Vermont Institutional Review Board and deemed exempt category 2 of 45 CFR 46.104. Pilot study activities did not meet the federal regulatory definition of human subjects research and therefore did not require IRB review and approval.

### 2.1 Data collection methods

Data collection involved stakeholder interviews, focus groups, and a pilot study. Between November 2022 and March 2023, 17 stakeholder interviews and 7 focus groups were conducted, each lasting approximately 45–60 min. Interviews and focus groups were held in person or virtually via Zoom. Information consent was obtained before all sessions, which were audio or video-recorded and transcribed by a third-party professional transcription service.

### 2.2 Stakeholder interviews

Stakeholder interviews were conducted with representatives from state agencies and local healthcare, education, social service organizations such as senior housing and after-school programs serving rural communities. These organizations were involved in COVID-19 communication, testing, and emergency response efforts. Participants were recruited using a snowball sampling approach.

The interview protocol included information on dissemination strategies for public health messaging, accessibility and availability of COVID-19 testing, and effectiveness. Key topics included how rural residents typically access health information, new or alternative information sources used during the pandemic, discrepancies in the information provided, and communication strategies employed by stakeholders to effectively reach the public.

### 2.3 Focus groups

To identify organizations in isolated, small, and large rural communities, the study team used the rural–urban commuting area (RUCA) codes (39, 40). Collaboration with community organizations supported the recruitment of rural residents for focus groups. Focus group participants included adults living in rural areas. Some groups were selected to reflect a range of perspectives across age and caregiving roles, and to capture communication experiences across different levels of digital access and health information needs, such as residents in senior congregate housing and parents of school-aged children.

Focus group discussions explored participants’ trusted sources of health information, their decision-making processes for COVID-19 testing, and facilitators and barriers they encountered in accessing testing. These discussions provided insights on how rural communities navigated the information landscape during the COVID -19 public health crisis. Participants in the focus groups were offered a $30 gift card for their participation,

### 2.4 Implementation strategy-pilot study

**Figure 2:**
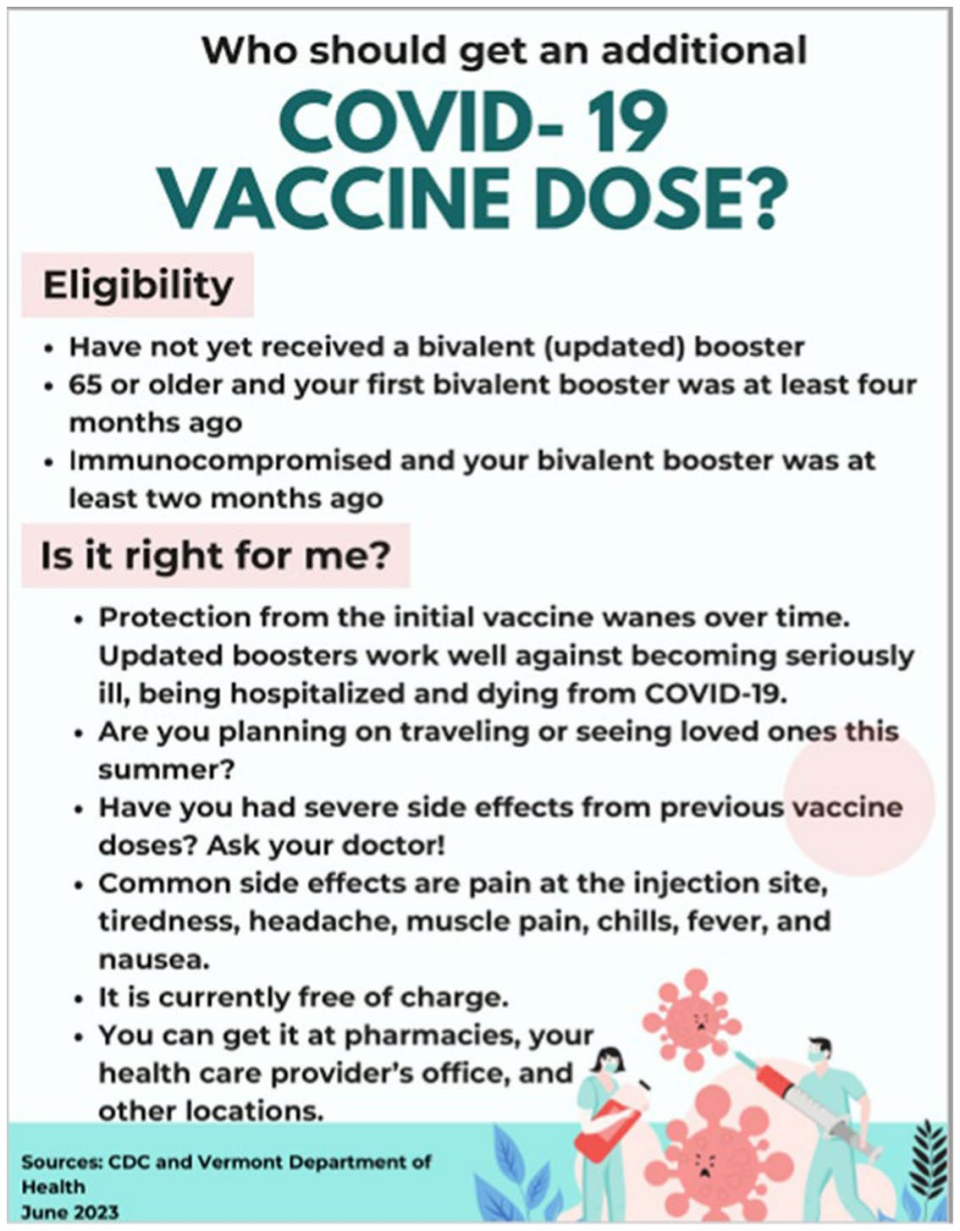
Flyer for pilot study.

A pilot study was conducted with older adults in rural congregate housing to gather direct feedback on health communication materials. This pilot study was designed specifically to test how feedback from the target population could influence the refinement of health communication strategies. Working closely with a community stakeholder, we created a flyer based on the stakeholder’s preferences for large fonts and images, with a focus on COVID-19 vaccine updates. After distributing the flyers, we used a brief paper survey to assess the effectiveness of the materials (Figure 2).

The survey gauged participants’ understanding of the information, their preferred methods of receiving evaluating, and assimilating health information, and their confidence in making health decisions based on the materials. Feedback from this pilot study highlighted areas for improvement in how the messages were communicated, particularly around clarity and presentation. This information was integrated into the broader analysis of public health messaging as part of the “Feedback” element of the PHERCC framework. This helped to refine the approach for future communications and ensured that the materials were tailored to the needs of the intended audience.

### 2.5 Data analysis

Thematic analysis was employed to analyze data from stakeholder interviews, focus groups, and the pilot study. The study team reviewed transcripts for accuracy and developed a list of *a priori* codes based on the six elements of the PHERCC framework: Evidence, Initiator, Channel, Publics, Message, and Feedback (Figure 1). These elements formed the foundation of the codebook, ensuring consistency in data organization and interpretation. All analyses were conducted using QSR International (64).

## 3 Results

### 3.1 Stakeholder characteristics

Stakeholder representation was diverse, encompassing both statewide entities and community-level organizations. The seventeen (17) statewide stakeholders included representatives from the Department of Health, the Medical Center, and the Association of Hospitals and Health Systems. Community-level stakeholders were drawn from Community Health Teams, Senior Housing Programs and After-School Programs. Additionally, the representative from Emergency Medical Services bridged both statewide and local perspectives.

### 3.2 Focus group demographics

The focus groups consisted of 30 participants, primarily white (86.7 percent) and female (76.7 percent), with nearly half aged 65 and older (46.7 percent). Most participants had health insurance (96.7 percent), had been tested for COVID-19 (96.7 percent), and lived in rural areas, with 40 percent living in isolated small rural regions. Educational attainment was diverse, with 63.3 percent having some college education or higher, 50 percent were currently employed, and 43.3 percent were retired (Table 1).

**Table 1:** Focus Group Participant’s Sociodemographic Data

**Variable**	**Categories**	**Percent (N=30)**
**Age (years)**	18-25	0
	26-34	6.7
	35-54	26.7
	55-64	13.3
	65+	46.7
**Tested for COVID-19**	Yes	96.7
**Ethnicity**	Hispanic, Latino, or Spanish	0
	Not Hispanic, Latino, or Spanish	93.3
**Race**	American Indian or Alaska Native	0
	Black or African American	0
	More than one race	6.7
	White	86.7
**Gender**	Man	23.3
	Woman	76.7
	Non-binary/ Genderqueer/ Gender nonconforming	0
**Education**	Less than high school	10
	High school graduate	10
	Some college or associate's degree	33.3
	Bachelor's degree (BA, AB, BS, BBA)	30
	Graduate degree (Master's, Professional)	16.7
**Insurance**	Yes	96.7
	No	3.3
**Have Kids<18**	Yes	60
	No	40
**Employment Status**	Disabled, permanently or temporarily	3.3
	Looking for work, unemployed	0
	Retired	43.3
	Stay-at-home Partner / Spouse	0
	Student	0
	Working now	50
**Rural-Urban Location** *	Large Rural	23.3
	Small Rural	36.7
	Isolated Small Rural	40

*RUCA = Rural Urban Commuting Area Codes, reference: https://www.ers.usda.gov/data-products/rural-urban-commuting-area-codes/

### 3.3 Implementation strategy-pilot study participants

Nine rural older adults also participated in the pilot study evaluating health communication materials.

### 3.4 Evidence: grounding communication in facts and data

Stakeholders emphasized the importance of ensuring that public health messaging adhered to the best available evidence, even when that evidence was incomplete or rapidly evolving. Guided by the principles of Crisis and Emergency Risk Communication, stakeholders prioritized transparency by clearly communicating what was known, what remained uncertain, and the steps being taken to address those gaps. As one stakeholder explained, *“the main thing and most important thing for us was being that go-to credible source and following the principles of the Crisis and Emergency Risk Communication Team, which is be out there first, if you can, be right, be timely, be empathetic, give the people what they need and tell them when you are going to know more, admit what you know, admit what you do not and what you are doing to find out”.*

The dynamic nature of the pandemic and the initial scarcity of evidence presented significant challenges for public health messaging. Stakeholders acknowledged the frustration when authorities had to admit gaps in knowledge or make difficult decisions. As one stakeholder noted, *“fear and then, of course you are frustrated when public health is saying we do not really know, or we do not have enough of that, and we have to make decisions on who gets what.”* Early guidance, such as using “face coverings” instead of “masks,” reflected both the limited evidence and the evolving understanding of the virus. As evidence emerged, messaging adapted, with campaigns simplifying recommendations, like “masks on faces, six-foot spaces, and uncrowded places,” to promote harm reduction.

The scarcity of resources, such as testing supplies, required tough decisions early on. As one stakeholder explained, *“in the beginning it wasn’t available. It was like if you were part of an epidemiological investigation for COVID there were very few testing supplies and it was very narrow.”* Despite these challenges, stakeholders emphasized that transparency about the evolving situation and decision-making was important to maintaining public trust.

Stakeholders frequently referred to expert opinions and guidelines from national entities, such as the CDC, as foundational to their messaging strategies. This reliance ensured alignment between public health communications and the best available evidence. Reviewing and revising communication scripts was a routine practice to validate that message accurately reflected expert guidance and emerging knowledge. As one stakeholder noted, *“My role was to look at the scripts. Make sure the information we were putting out lined up with what we are hearing from our experts.”*

### 3.5 Initiator: establishing credibility and trust

The Department of Health was the primary regional initiator of the COVID-19 communication efforts, responsible for creating, managing, and disseminating public health messaging to both the public and other key stakeholders. The department played a central role in organizing and distributing critical information, including testing guidance, quarantine protocols, and health safety measures, ensuring that all communication came from a unified source. As one stakeholder said, *“All public communication through the Department of Health related to COVID messaging came through us and our team.”* This centralized approach allowed the department to become the go-to and trusted source of information in a rapidly changing landscape.

The Department of Health worked quickly and effectively to establish systems for distributing messages, including press releases, social media posts, and updates to the public. The team was responsible for adapting the messaging as new information became available, ensuring that the public received timely, accessible, and accurate updates. As one stakeholder said, *“We were learning about COVID as we were sharing it.”* The department’s team was also closely involved in the website updates, ensuring that the online resources were continually refreshed to reflect the evolving situation.

As the pandemic progressed, the department’s outreach grew significantly, particularly on social media. The rapid increase in followers highlighted the increasing public demand for information directly from the Department of Health. As one stakeholder mentioned, *“You can look at the increases in our activity on social media, like the people that were following us went from 12 to 12,000 in a really short span”.*

A key aspect of the department’s ability to respond effectively was the establishment of a new communications infrastructure. This infrastructure was created by leveraging existing human resources and expertise within the department, bringing together scattered communications experts to work cohesively and manage the flood of inquiries from the public. According to one stakeholder, *“We took all that knowledge and human power and put together an infrastructure that responded to a ridiculous amount of questions. Empathy and honesty were at the middle of what we were trying to do on the … team.”* This new infrastructure allowed the department to answer questions, distribute information, and adapt strategies as circumstances evolved.

In addition to its direct communication efforts, the Department of Health collaborated with a variety of stakeholders to amplify its messaging. This included partnerships with schools, healthcare facilities, municipalities, and other community organizations. The department also worked with specialized teams, such as the Public Information Team and Epidemiology Team, to ensure that information was consistently and accurately distributed. As one stakeholder said, *“We had weekly meetings with the hospital, colleges, and municipalities there was a web of overlap that we would utilize to push the message out”*.

Stakeholders emphasized the value of pre-established communication systems and advocated for clearer alignment and collaboration between state and national public health authorities in future responses. This reinforces the need for strong centralized communication channels that can adapt quickly to evolving public health needs.

### 3.6 Channels: selecting the right platforms for outreach

The communication strategies employed during the pandemic involved a variety of methods, with media outlets, digital platforms, printed materials, community networks, and direct communication playing key roles in ensuring information reaches diverse segments of the population. Media outlets were central to keeping the public informed throughout the pandemic. Press briefings by the State governor and public health officials were especially critical in providing accurate, up-to-date information. One stakeholder described how the governor’s press conferences were effective: *“I think one of the things that I actually thought our governor did well was his press conferences, he made sure that he had access… and there was the combination of … giving the scientific information.”* A focus group participant echoed this sentiment, saying, “*I think in the beginning when the governor had all these meetings. Those were very helpful to me. They were very informative*.” These briefings held regularly, were described by both stakeholders and focus group participants as a reliable and consistent information during the early stages of the pandemic.

In addition to press conferences, radio broadcasts also played a significant role in disseminating information. One stakeholder mentioned, *“Biweekly radio interview broadcasts with a chief medical officer of a hospital for updated info on COVID.”* These broadcasts allowed for a wider audience, ensuring that people in the community stayed informed through an accessible channel.

Digital platforms became increasingly important as the pandemic progressed. Social media and email updates were key to reaching the public in a timely manner. One stakeholder explained, *“We used a mix of social media posts, emails, and our website to ensure everyone was informed. It was important to keep information consistent across all platforms to reach as many people as possible, especially those who relied on different methods of communication.”* Social media allowed for real-time communication, while email provided a way to disseminate more detailed guidance and updates.

A particularly useful platform was Front Porch Forum, a local social media tool that allowed for targeted communication with different communities. As one stakeholder noted, *“The biggest thing that …uses is this site called Front Porch Forum, which you guys probably know of. But honestly, any time we talked to the department of health’s communications team, the first thing they would do is put it on Front Porch Forum.”* This platform was used to share public health updates.

While digital platforms were increasingly important, printed materials remained essential, particularly for those with limited access to technology. Flyers, newsletters, and roadside signs were commonly used to distribute important information. One stakeholder explained, *“We put out flyers to the hotels where the hotel guests who are experiencing homelessness were staying, so they knew how to access transportation to get to the testing.”* Printed materials were placed in public spaces such as healthcare facilities, schools, and community centers, ensuring that people encountered them in places they frequented.

Community networks were important in ensuring that people without internet access or reliable technology could still receive important information. Stakeholders in rural areas emphasized how community-based efforts and local partnerships made this possible. According to a focus group participant, *“we have a local health center in our town, so that’s where I would rely on getting factual up to date information or my questions answered*.” One stakeholder described how they distributed hardcopy materials to students in a rural area, saying, *“In …, for example, information was sent home via hard copies… information was sent home once a week via a newsletter*”.

Additionally, community members and organizations acted as conduits for information. One stakeholder highlighted how schools and local community health teams used their networks to share updates: *“We also use all of our email … platforms to help share information with families about COVID spread and how to reduce that spread.”* A focus group participant mentioned “*I have kids in school, so a lot of the information was coming through email and updates from school*.” Another focus group participant reiterated, *“so if I have a question about COVID or medical, I start with her at (community partner), and then they direct me as needed.*” This grassroots, community-centered approach helped ensure that information reached everyone.

Lastly, direct communication was vital for personalizing and clarifying information for community members. Stakeholders frequently used phone calls, one-on-one visits, and in-person meetings, when possible, to ensure individuals had access to the information they needed. For seniors and those with specific needs, these personal interactions were an essential means of ensuring they understood public health guidelines. Guidance over the phone was an important strategy for supporting individuals in navigating available resources. As one stakeholder explained, *“I would encourage them to call the Department of Health or call the COVID call center. I would look things up on the computer and share them over the phone, or I would encourage them to call the other resources”*.

### 3.7 Publics: identifying and engaging target audiences

Trust in the sources of health information played a crucial role in how messages were received and acted upon by different groups. Stakeholders observed that familiarity with the messenger significantly influenced the effectiveness of communication. Locally situated Emergency Medical Services and COVID testing facilities were often relied upon to disseminate information, resharing posts from trusted sources like the Department of Health and the CDC. One stakeholder reflected, *“I always remember that early on … ambulance would share the post from the CDC or Department of Health. We would broadcast that. We would also educate our staff… but it was primarily we would just reshare posts, rarely make our own posts. We always took it from a more senior organization and official organizations.”*

Stakeholders noted that relying on familiar, trusted organizations helped build public confidence in the credibility of information. This approach reinforced the reliability of messages and increased the likelihood that guidance would be followed, especially during times of crisis. They also emphasized that using local voices to amplify public health messages strengthened familiarity and trust, supporting more effective communication within communities.

Public health stakeholders recognized the need to adapt communication strategies to address barriers and maximally reach community specific groups effectively. Different populations had distinct preferences and levels of access to information, with older adults in rural areas being particularly disadvantaged. Many lacked access to digital platforms and were not involved with community organizations, such as schools, that might otherwise disseminate health updates. As one stakeholder highlighted, *“So the older people aren’t getting the information because they do not have access to the Internet, and they are not affiliated with the schools, so they are not getting the information from the schools. So that’s a definite gap in our area.”* Community partnerships were a key strategy in filling gaps in access. For example, one stakeholder shared, *“Putting the email out allowed our local Council on Aging to put that information in with the Meals on Wheels deliveries.”* This ensured that older adults and those with limited internet access received critical updates.

Traditional media, such as newspapers and TV commercials, also proved vital in reaching rural populations. One stakeholder noted, *“Ironically, one of the ways that we found that we were able to reach older people was to put it in the old-fashioned newspaper.”* However, while TV commercials provided general information, they often lacked the specificity needed to address the unique challenges of rural communities. As another stakeholder explained, *“The … commercials were great for information in a broad sense, but they were not good for very specific information for a population that was vulnerable and did not have access or ability to get testing as readily.”* To address these challenges further, stakeholders implemented additional methods, such as phone lines and voicemail systems, to provide timely updates on clinic hours and testing locations.

Language barriers and social factors played a critical role in shaping how different groups understood and responded to public health messages. Stakeholders recognized early in the pandemic that non-English-speaking communities were not being adequately served, particularly following outbreaks in these populations. In response, stakeholders prioritized translating messages into multiple languages and worked closely with community leaders to improve communication. One stakeholder emphasized the importance of inclusivity, noting, *“Translating messages into 14 languages and working with community leaders helped us reach more diverse groups.”* This shift demonstrated the growing understanding that public health messaging must be culturally sensitive and accessible to ensure all groups, regardless of language, have access to accurate health information.

### 3.8 Message: crafting clear and effective communication

Public health authorities faced significant challenges in crafting and delivering clear and consistent messaging throughout the evolving COVID-19 pandemic. Rapid changes in public health guidance such as shifting recommendations on mask-wearing created confusion, as individuals often retained the first message they encountered. Stakeholders acknowledged that frequent message revisions risked undermining credibility.

One stakeholder explained, *“There was a delay in information. If the CDC came out with something, it took a minute for the Department of Health to turn that into their materials and to get that out to the community.”* This delay was compounded by the speed at which guidance evolved, resulting in outdated materials circulating. Also, another stakeholder described the challenge, saying, *“I spent so much time printing things and then going around to testing sites and taking the pamphlets away and being like, ‘No, this is not up to date anymore. Stop giving it to patients.’”*

To address these challenges, public health teams prioritized simplifying and streamlining messages to ensure clarity. Core prevention actions, such as maintaining physical distance, wearing masks, and staying in safe spaces, were emphasized. This harm-reduction approach encouraged incremental safety improvements rather than perfection.

To maintain consistency amidst evolving guidance, public health authorities relied on basic but effective tools such as partner toolkits, branded visual materials, and online resources. These materials were designed to ensure a cohesive “COVID look” that reinforced the legitimacy of messaging. One stakeholder highlighted the collaborative nature of this effort, explaining, *“We created some of our own materials or agreed to use the CDC materials and distributed those as well. There was a lot of work at that group level, making sure we were putting out the same message and being cohesive in our community so as to not be confusing and to be clear”*.

Weekly calls with point people at testing sites also helped maintain consistency. A stakeholder described the process: *“I had regular weekly calls where people could call in, and we would go through things I was hearing from my end, things they were hearing from their end. It was about making sure everyone was clear about what information they were providing.”*

Stakeholders also emphasized ensuring consistent and up-to-date messaging across all community touchpoints. As one community health stakeholder explained, *“So part of our role, my role as a community health team lead, was just to make sure all of those resources where people do touch in, have the same messaging, have that up to date schedule of where you can get testing and know how they can access that.”* Stakeholders ascertained that this coordination maintained public confidence and ensured that individuals could access reliable, actionable information.

### 3.9 Feedback: adapting and improving through public response

Public feedback during the pandemic was critical in shaping communication strategies and refining messages. Stakeholders identified fear and uncertainty as dominant public sentiments, influencing how messages were crafted to resonate with various groups. Concerns ranged from personal safety to skepticism about government transparency. *“The messages we were getting from the public overwhelmingly were: How do I protect myself? Fear of resource scarcity and frustration. But it was all based on fear.”* Recognizing these concerns, stakeholders adapted messaging strategies, including debranding materials and collaborating with local partners to foster community trust. In rural areas, stakeholders noted that tailoring communication to local contexts improved message reception, highlighting the importance of community-driven approaches. They emphasized that using trusted messengers and familiar communication channels, such as town newsletters or community organizations, helped ensure that messages were relevant and credible. This approach was viewed as especially important in areas with limited internet access. Focus group participants noted that messages shared by known local figures felt more reliable. As mentioned by one focus group participant, “*I trust [Community partner] first, I go to her because you cannot trust the Internet”*.

Stakeholders encountered significant communication challenges, including discrepancies between official information and that provided by external sources like news media and pediatricians. Additionally, translation services were initially limited, complicating outreach to the State’s linguistically diverse communities. Efforts were made to create pre-translated templates for rapid communication, yet real-time translation during emergencies remained a challenge. Scalability also posed issues as the call center expanded from one operator to 65 staff members to manage the surge in inquiries.

Stakeholders employed data-driven methods to continuously refine messaging based on public feedback. Regular analysis of website traffic, social media engagement, and frequently asked questions guided adjustments to communication strategies. *“We would look for trends across all communication channels every day and make sure that those answers were reflected in all the different ways people would receive information, including press conferences.”* Familiar channels, like local newsletters and community organizations, significantly boosted engagement, with social media followers increasing from 6,000 to 25,000 in one month. One focus group participant shared, “*I use the Department of Health’s website a lot just to try and keep up with all the changes*.” Despite evolving health guidelines and complex testing systems, stakeholders remained dedicated to improving communication. Public feedback on barriers to testing access led to acknowledgments of system limitations and the pursuit of user-friendly solutions. These iterative improvements to communication align with the drop-loop model’s emphasis on adjustment and planning during the stabilization and recovery phases of a crisis (29).

### 3.10 Implementation strategy results

Nine participants completed surveys, unanimously agreeing that the health information provided was easy to understand, presented in their preferred format, increased their knowledge about COVID-19 topics, and improved their confidence in making health decisions for themselves or their families. Additionally, all participants expressed interest in receiving future health information in a similar format. The community partner involved in the project reported high satisfaction with both the content and delivery method, expressing interest in future collaborations.

## 4 Discussion

This study underscores the critical role of transparent, evidence-based communication in public health crises, particularly during the COVID-19 pandemic. Stakeholders emphasized the need for clear and consistent messaging, even when scientific knowledge was incomplete, aligning with the Public Health Emergency Risk and Crisis Communication (PHERCC) framework, which advocates for timely, transparent communication to maintain public trust and promote compliance in the face of uncertainty (41, 42).

The PHERCC framework aligns closely with the stages of the drop-loop model, which outlines how public health crises unfold, from a baseline to acute crisis and ultimately to recovery and development. Our findings demonstrate how risk communication strategies varied across these phases. During the acute crisis, for instance, stakeholders emphasized the urgency of providing evidence-based and rapidly evolving messages to maintain public trust. As the crisis stabilized, the emphasis shifted toward refining messages, integrating public feedback, and addressing access and equity, reflective of the Feedback and Publics domains of PHERCC. This dynamic adjustment process illustrates the value of applying the drop-loop model in tandem with PHERCC to guide real-time communication decisions across all stages of a public health emergency. The PHERCC domains align with key phases of the drop-loop model; for example, Message and Channel are central during the acute crisis phase, while Feedback and Publics are essential during stabilization and recovery.

Transparent communication, especially about evolving guidelines such as mask-wearing and social distancing, was essential to maintaining public trust (43). This approach, which prioritizes being “first, right, and timely,” provided the public with a clear understanding of what was known and what remained uncertain. This level of transparency was essential in managing public expectations and in fostering trust, particularly as public health messages evolved in response to new evidence. The shifting guidance surrounding face coverings, for instance, highlighted how public health recommendations had to be adjusted as more information about COVID-19 emerged. This adaptability was a key feature of effective messaging, as it demonstrated both responsiveness to new scientific insights and a commitment to mitigating harm. Maintaining consistency in messaging posed challenges due to the rapid changes in public health guidance. Stakeholders responded by simplifying core messages and using tools like partner toolkits and regular updates to ensure clarity and minimize confusion (44–47). Public feedback played a pivotal role in refining communication strategies, addressing public concerns, and building trust (48–50). The importance of community collaboration was evident, with stakeholders leveraging local platforms, such as online community forums, to counter misinformation and enhance message credibility.

Our research highlights the importance of adapting communication strategies to diverse populations. Stakeholders noted challenges in reaching rural older adults, often without digital access, and used traditional media like newsletters and print ads to overcome these barriers, as noted in previous literature (51, 52). Additionally, language barriers were a key factor, with translated materials and partnerships with community leaders ensuring more effective outreach, particularly to non-English-speaking communities (53–56).

These findings highlight how rural context fundamentally shapes both the delivery and effectiveness of risk communication during public health emergencies. Geographic isolation, limited broadband access, and a lack of formal communication infrastructure posed major barriers to timely information sharing. In response, rural communities relied heavily on informal networks, printed materials, and trusted local messengers to fill communication gaps. Stakeholders also described how strong local partnerships with schools, health centers, and community organizations helped tailor messages to specific needs and reach groups that might otherwise be left out. These adaptations point to the need for communication strategies that go beyond linguistic tailoring and are structurally adapted to the realities of rural settings (56).

Another key finding was the significance of trust in messaging sources. Local Emergency Medical Services and community organizations often relied on resharing messages from trusted sources like the CDC and Department of Health, which helped build credibility and foster community engagement (57–59). This approach was crucial, especially in crisis situations where familiarity with the messenger enhanced message effectiveness.

While much of this study focused on communication strategies that stakeholders considered effective, several key challenges were also identified, particularly within rural communities. A major issue was reaching older adults and others without reliable internet access, which limited the effectiveness of digital communication channels. Language barriers and the delayed availability of translated materials posed additional challenges for non-English-speaking residents. Moreover, rapidly changing guidance often led to confusion, with outdated printed materials and conflicting messages from various sources undermining public trust. The PHERCC framework helped bring these issues into focus by emphasizing the importance of tailoring messages to distinct publics, ensuring message clarity, and incorporating feedback loops. For example, the Feedback and Publics domains made it easier to identify how structural barriers, such as limited digital access or a lack of local infrastructure, impacted the equitable delivery of risk communication.

The pilot project results emphasized the importance of community collaboration in improving health communication. Participants reported high satisfaction with clear, accessible information, which helped build trust and confidence in COVID-19 messaging (60, 61). These findings revealed the need for adaptive, inclusive communication strategies that prioritize trust, cultural sensitivity, and public feedback to ensure effective messaging during future health crises. Through this pilot project, practical approaches and health communication preferences for a specific population were identified. Collaborating with community partners was important in ensuring the appropriateness, inclusivity, and effectiveness of health information. This collaboration enhanced comprehension, building trust in the messengers, and fostered greater comfort and confidence in the shared information (62, 63).

A limitation of this study is the reliance on qualitative data from a limited sample of stakeholders, which may not fully capture broader perspectives or experiences. The retrospective nature of the research may also introduce recall bias. Additionally, because this study focuses on rural communities in Northern New England, findings may not be generalizable to urban settings or other regions. However, many of the communication challenges identified, including infrastructure limitations, access barriers, and the importance of trusted local messengers, are also present in other rural and underserved areas. As such, the insights presented here may be relevant beyond the immediate study region. Future studies could assess long-term impacts of communication strategies on public trust and behavior.

## 5 Conclusion

In conclusion, this study emphasizes the need for clear, adaptive communication strategies in public health emergencies, as highlighted during the COVID-19 pandemic. By following the PHERCC framework, stakeholders shared that transparent, evidence-based messaging, alongside trusted local sources, is vital to building and maintaining public trust. Challenges such as reaching uniquely vulnerable populations, particularly older adults with limited digital access, were addressed through traditional media and community partnerships. Consistent messaging, adapting to evolving guidance, and public feedback helped refine strategies to ensure clarity and inclusivity. These insights foster the importance of community-driven communication and the need for continued investment in communication infrastructure to ensure effective responses in future health crises.

## Data Availability

The original contributions presented in the study are included in the article/supplementary material, further inquiries can be directed to the corresponding author.
